# Hoffmann’s syndrome in the differential work-up of myopathic complaints: a case report

**DOI:** 10.1186/s13256-023-04184-6

**Published:** 2023-10-31

**Authors:** Sabine Winter, Bianka Heiling, Niklas Eckardt, Christof Kloos, Hubertus Axer

**Affiliations:** 1 Department of Neurology, Jena University Hospital, Friedrich Schiller University, Am Klinikum 1, 07747 Jena, Germany; 2 Clinical Scientist Program OrganAge, Jena University Hospital, Jena, Germany; 3 Department of Radiology, Jena University Hospital, Friedrich Schiller University, Jena, Germany; 4 Department of Internal Medicine III, Jena University Hospital, Friedrich Schiller University, Jena, Germany

**Keywords:** Hoffmann’s syndrome, Hypothyroidism, Myopathy, MRI, Muscle biopsy, EMG

## Abstract

**Background:**

Hoffmann’s syndrome is a rare form of hypothyroid myopathy in adults, which is mainly characterized by muscular weakness and muscular pseudohypertrophy.

**Case presentation:**

We report about a 61-year-old Western European man with myalgia, myxedema and pseudohypertrophy of the calf muscles. Laboratory tests revealed significantly elevated thyroid stimulating hormone (TSH) and creatine kinase (CK). Muscle MRI showed muscular hypertrophy of the lower limbs, but no signs of myositis or myopathy (no gadolinium enhancement, no edema, no fatty degeneration). In addition, electromyography (EMG) detected spontaneous activity. After the beginning of thyroxin-therapy it took six months until the muscle weakness improved and the myalgia regressed.

**Conclusions:**

Here, we focus on diagnostic routines and typical findings to differentiate Hoffmann’s syndrome from other myopathies. Clinical hallmarks of Hoffmann’s syndrome are pseudohypertrophy and weakness of the calf muscles in combination with elevated CK and elevated TSH. EMG is well suited to detect the involvement of the muscles and muscle MRI helps to differentiate it from other myopathies. Hoffmann’s syndrome is a rare myopathy due to hypothyroidism and plays a role in the differential diagnosis of myopathic complaints even if hypothyroidism has not been detected before.

**Supplementary Information:**

The online version contains supplementary material available at 10.1186/s13256-023-04184-6.

## Introduction

The thyroid hormone is essential for the cell metabolism of all human organic systems. One of the prevalent malfunctions of the thyroid gland is primary hypothyroidism [1], which can be associated with a rare muscular manifestation, i.e. Hoffmann`s Syndrome in adults and analogously Kocher-Debré-Sémélaigne syndrome in children [2]. Hoffmann`s Syndrome is characterized by (proximal accentuated) weakness of limb muscles with an increase of muscle mass (pseudohypertrophy), muscle stiffness and cramps. It occurs most often in male adults with longstanding untreated hypothyroidism [2, 3]. Muscular pseudohypertrophy is a very rare phenomenon, which predominantly involves the calf muscles (especially the gastrocnemius muscles) [4]. The syndrome was first described in 1896 by the German neurologist Johann Hoffmann (1857–1919) [5, 6].

In addition to classic hypothyroidism-associated symptoms such as somnolence, cold intolerance, depressed mood, and myxedema, muscular symptoms such as exercise intolerance, myalgia, cramps, stiffness, and myoedema are common [7, 8]. It is known that hypothyroidism may be associated with an impairment of peripheral nerves, neuromuscular junction, and muscular fibres [9].

Indicative for hypothyroid myopathy (HM) is the finding of elevated thyroid stimulating hormone (TSH) in combination with elevated creatine kinase (CK) or lactate dehydrogenase (LDH) for at least 2 weeks [10]. The leading cause for HM is Hashimoto thyroiditis [9].

The underlying pathophysiology for HM is still not definitely known. One pathomechanism may be an alteration of glycogen metabolism and oxidative phosphorylation in the muscle [11]. Serum thyroid hormone T3 is one of the major hormonal regulators of glucose metabolism in mitochondria of the skeletal muscle. Thus, patients with hypothyroidism have a reduced glycogenolytic activity in the muscles, which can be proven by low levels of acid alpha-glucosidase in muscle cells and accumulation of glycogen deposits [11]. In addition, the actin-myosin unit is altered. Hypothyroidism leads to an atrophy and loss of type 2 muscle fibres (fast twitch and fast contraction fibres) and hypertrophy of type 1 fibres (slow twitch and slow contraction fibres) [12–16]. The third mechanism is the change of the glycosaminoglycan metabolism due to a higher urinary excretion of glycosaminoglycans in hypothyroid patients [17].

The treatment of HM is a replacement therapy with thyroid hormone (LT_4_). However, clinical recovery may be incomplete so that some patients have persistent myopathic symptoms over years. The variability of response to LT_4_ treatment is supposed to depend on the severity of previous muscle damage and the age of the patient [18].

## Case presentation

**Fig. 1:**
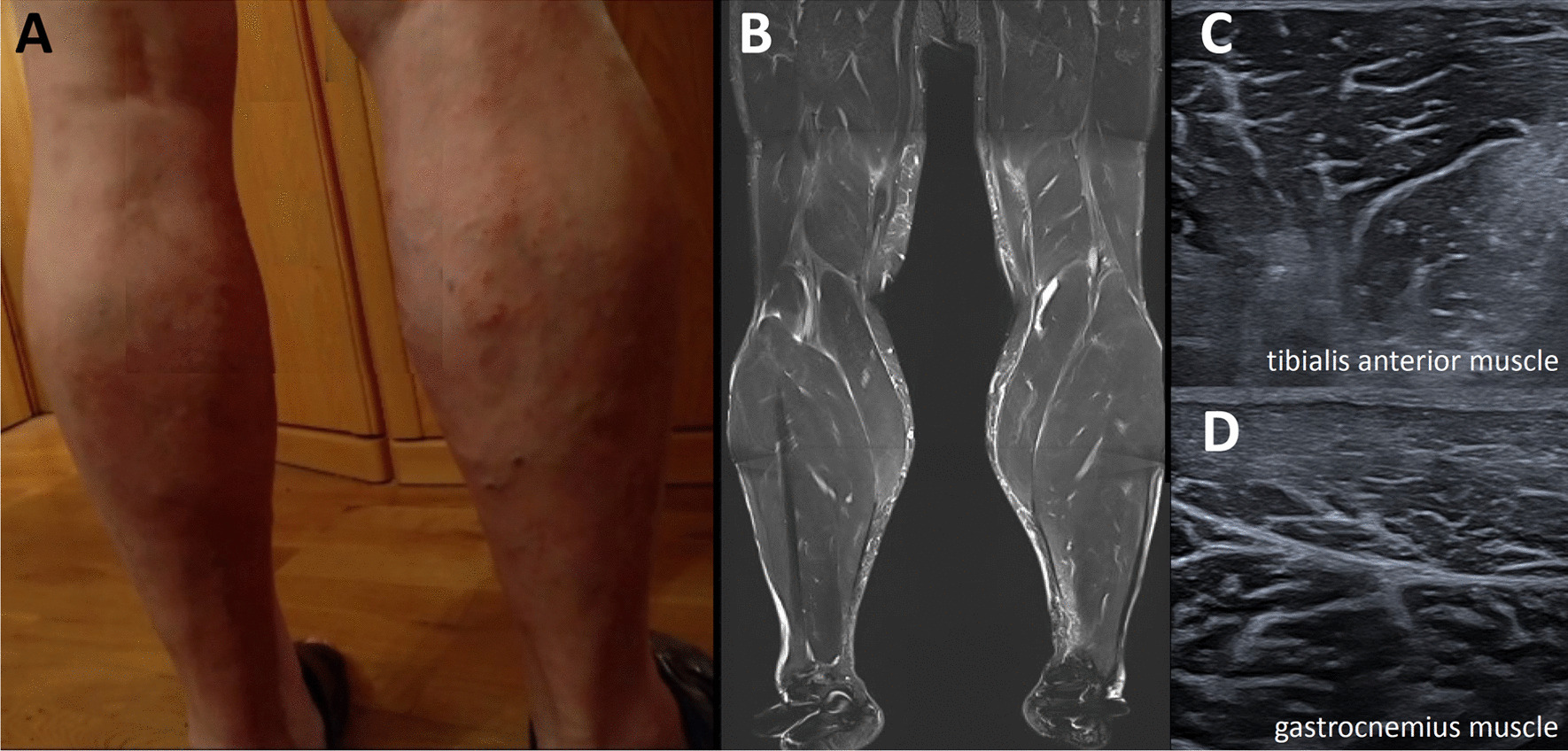
Calf muscles of the patient. **A** Photograph. **B** Muscle MRI. **C** and **D** Muscle ultrasound

A 61-year-old Western European man applied to our neurological outpatient clinic complaining about swollen hands for 8 weeks, myalgia dependent on physical activity in the lower and upper limbs for 3 months, general muscular weakness, and a hoarse voice without dysphagia. The clinical neurological examination revealed mild dysarthria, hypertrophy of bilateral calf muscles (Fig. 1A) without tenderness on palpation, and hypo-/areflexia with pallhypesthesia of the lower limbs. In addition, the patient reported about paresthesias in the first three fingers and the radial forth finger of the right hand with a positive Phalen’s sign suggestive of carpal tunnel syndrome. Except for mild edema of the limbs the cardiovascular and respiratory system showed no abnormality.

CK was significantly elevated (64 µmol/l*s, normal < 3.2), and electromyography (EMG) showed spontaneous activity (positive sharp waves and fibrillation potentials) in the muscles (right tibialis anterior and left gastrocnemius muscle) but no signs of chronic denervation and no myopathic changes.

Thus, the patient was subjected to our neurological ward at Jena University Hospital. Nerve conduction study showed bilateral carpal tunnel syndrome but no signs of polyneuropathy. Magnetic evoked potentials were normal. The most important finding was an elevated TSH up to 30.5 mU/l (normal range: 0.27–4.20 mU/l), the level of T3 was under 1.50 pmol/l (normal range 3.10–6.80 pmol/l) and the T4 level under 1.30 pmol/l (normal range 12.00–22.00 pmol/l). Thus, manifest hypothyroidism was diagnosed. Anti-thyreoglobulin-antibodies (5671.0 U/ml, normal < 60 U/ml) and anti-TSH-receptor-antibodies (5.4 U/ml, normal < 1.8 U/ml) were increased, and Hashimoto thyroiditis was diagnosed [19]. Myoglobin was elevated to 248 µg/l (normal range 28–72 µg/l). Blood count showed a mild normocytic, normochrome anemia, the liver enzymes were elevated (ALAT up to 1.94 µmol/l*s, normal < 0.83 µmol/l*s; ASAT up to 2.75 µmol/l*s, normal < 0.85 µmol/l*s). Renal function did not show any significant abnormalities. Cerebrospinal fluid showed cytoalbuminologic dissociation without evidence of infection. Rheumatologic parameters, hepatitis serology, lipid profile, and vitamins revealed no pathologic findings.

Electrocardiogram, cardiac ultrasound and pulmonary function testing demonstrated no pathologic findings. Magnetic resonance imaging showed a muscular hypertrophy of the lower limbs (Fig. 1B), but no signs of myositis or myopathy (no gadolinium enhancement, no edema, no fatty degeneration).

Consequently, replacement therapy with levothyroxine (LT_4_), was initiated starting with 75 µg per day. Myopathic symptoms improved over six months but there was no complete remission of the symptoms. Especially the bilateral calf hypertrophy persisted. Signs of chronic denervation evolved in EMG with elevated amplitudes of muscle unit action potentials (right and left tibialis anterior, left gastrocnemius, and right vastus lateralis muscles), positive sharp waves were still detectable in the right tibialis anterior muscle. Muscle ultrasound revealed a normal to slightly increased echogenicity of the calf muscles (Fig. 1C). The patient reported about a good clinical recovery of muscle strength and decreased myalgia. CK and myoglobin decreased significantly (Table 1).

**Table 1:** Change of laboratory parameters over time

Period	Thyroxine T_4_	Thyroid-stimulating hormone	Creatine kinase CK	Myoglobin
Before treatment	< 1.00 pmol/l	30.5 mU/l	64.01 µmol/l*s	248 µg/l
After treatment (6 months)	–	3.16 mU/l	4.16 µmol/l*s	63 µg/l
Normal range	12–22 pmol/l	0.27–4.20 mU/l	< 3.20 µmol/l*s	28–72 µg/l

## Discussion

The prevalence of manifest hypothyroidism varies between 0–7% in the USA and 0–5% in Europe [20]. Decompensated hypothyroidism is a rare condition, with an incidence of 0.22–1.08 cases per million patients per year with women over 60 years of age being most frequently affected [21]. Hoffmann’s syndrome with its characteristic symptoms of muscular (pseudo-) hypertrophy is a rather rare manifestation of hypothyroidism [22].

We collected 21 case reports [3, 4, 18, 23–40] describing 23 patients with Hoffmann’s syndrome (Pubmed, search for: “Hoffmann syndrome hypothyroidism”, 08-25-2022). 20 of the patients were male and three were female with a mean age of 36 years (standard deviation 12.4) which demonstrates a clear male predominance of the syndrome in contrast to the female predominance in decompensated hypothyroidism.

Relative elevations of CK and TSH of the reported patients were calculated by division of each individual measurement by the largest normal value reported in the case reports because normal reference values differ between laboratories. Mean elevation of CK was 19.8fold (standard deviation 14.2) and mean elevation of TSH was 18.5fold (standard deviation 13.6). In 18 cases neurophysiological examinations were described, 6 case reports described muscle biopsy results and 3 described results of muscle MRI. The details are shown in Additional file 1.

Hoffmann’s syndrome with its characteristic symptoms of muscular (pseudo-) hypertrophy is a rather rare manifestation of hypothyroidism [22]. Muscular symptoms as described by hypothyroid patients like myalgia, weakness, stiffness, cramps and easy fatigability are similar to symptoms of other myopathies. More specific to hypothyroidism-associated myopathy are weakness of proximal muscles, myxedema, delay in deep tendon reflex and development of muscle hypertrophy [41]. Hypothyroid myopathy can also lead to rhabdomyolysis [42] and can therefore potentially be life-threatening.

Although there are several case descriptions of Hoffmann’s syndrome in the literature there is no consistent concept for diagnostic work-up with typical findings. Therefore, this case report brings diagnostic routines and possible findings into focus especially to differentiate Hoffmann’s syndrome from other myopathies.

The clinical hallmark of Hoffmann’s syndrome is the pseudohypertrophy of calf muscles as shown in our case and in all reported cases as well. In addition to elevated CK as sign of muscular impairment and elevated TSH levels as expression of hypothyroidism the differential workup of myopathies clearly points to the diagnosis of Hoffmann’s syndrome. Interestingly, there is a positive correlation between CK and TSH but no correlation with clinical severity [43].

Clinical neurophysiological methods were most often used to evaluate patients´ nerve and muscle function. Electromyography showed spontaneous activity such as fibrillation potentials and positive sharp waves in our patient as well as in five of the collected cases and complex repetitive (pseudomyotonic) discharges in three of the collected cases. In addition, low amplitude and polyphasic motor unit action potentials (MUAPs) were found, but also normal MUAPs were described.

Nerve conduction studies (NCS) showed carpal tunnel syndrome in our patient. It has been shown before that a considerable proportion of patients with untreated primary hypothyroidism have carpal tunnel syndrome [44] and hypothyroidism is a risk factor for carpal tunnel syndrome [45].

A study of 24 hypothyroid patients [45] showed clinical muscle weakness in 38%, signs of sensorimotor axonal neuropathy in 42%, and carpal tunnel syndrome in 29%. Short duration of MUAPs were seen in 33%, fibrillation potentials in 17%, and decreased nerve conduction velocity in 17% [46].

In six of the reported cases of Hoffmann’s syndrome muscle biopsy was performed. However, results were described very differently: increased hypertrophic fibres, muscle atrophy, increased nuclei, variation in fibre size, or muscle fibre necrosis. None of the reports found any signs of inflammation. One of the muscle biopsies was normal. In addition, type 1 fibre predominance, type 2 atrophy, glycogen accumulation and damaged mitochondria were described [22]. In addition, a case series of muscle biopsy results in eight patients with hypothyroid myopathy [47] found a dominance of type 2 fibres over type 1 fibres and 'core-like' structures, which disappeared after treatment. In contrast, no individual muscle fibre hypertrophy in the cases with Hoffmann's syndrome could be found [47]. Overall, morphologic changes described in hypothyroid myopathy appear to be largely nonspecific [22], but from the perspective of a differential clinical workup, a muscle biopsy may indicate muscular involvement.

The main finding in muscle MRI is T1 hypertrophy of the calf muscles (Fig. 1B). In addition, affected muscles may show patchy hyperintensities on T2-weighted images [3, 28, 29]. In contrast, muscle MRI shows no gadolinium enhancement, no edema, and no fatty degeneration in Hoffmann’s syndrome and may therefore be useful in the non-invasive differentiation from other myopathies. Muscle ultrasound in our case did not show specific changes (Fig. 1C, D).

There were some tests performed such as motor evoked potentials, when there are no upper motor signs or symptoms, and CSF studies when there are no cerebral of peripheral nerve symptoms. However, although they did not contribute to the diagnosis they were suited to exclude other differential diagnoses.

The therapy of choice is the substitution of thyroid hormones (levothyroxine, LT_4_) [48]. Most symptoms slowly regress over time after beginning LT_4_, but the electrophysiologic findings may persist [36, 38].

## Conclusion

Clinical hallmarks of Hoffmann’s syndrome are muscular weakness and pseudohypertrophy of the calf muscles in combination with elevated CK and elevated TSH. EMG is well suited to detect the involvement of the muscles and muscle MRI helps to differentiate it from other myopathies. Hoffmann’s syndrome is a rare myopathy due to hypothyroidism and plays a role in the differential diagnosis of myopathic complaints, even when hypothyroidism has not been diagnosed before.

### Supplementary Information

**Additional file 1: Table A1.** Case reports of Hoffmann’s syndrome.

## Data Availability

Data is contained within the article.

## References

[1] Vanderpump MPJ (2011). The epidemiology of thyroid disease. Br Med Bull.

[2] Sindoni A, Rodolico C, Pappalardo MA, Portaro S, Benvenga S (2016). Hypothyroid myopathy: a peculiar clinical presentation of thyroid failure. Review of the literature. Rev Endocr Metab Disord..

[3] Lee KW, Kim SH, Kim KJ, Kim SH, Kim HY, Kim BJ (2015). A rare manifestation of hypothyroid myopathy: Hoffmann’s syndrome. Endocrinol Metab (Seoul).

[4] Udayakumar N, Rameshkumar AC, Srinivasan AV (2005). Hoffmann syndrome: presentation in hypothyroidism. J Postgrad Med.

[5] Schmitt HP (2008). Größe im Schatten: In memorandum: Johann Hoffmann (1857–1919), neurologist and scholar. Nervenheilkunde.

[6] Hoffmann J (1896). Casuistische Mitteilungen aus der Heidelberger medicinischen Klinik (Prof. Erb). Dtsch Z Nervenheilk..

[7] Ramadhan A, Schondorf R, Tamilia M (2011). Rhabdomyolysis and peroneal nerve compression associated with thyroid hormone withdrawal in the setting of remnant ablation: review of the literature. Endocr Pract.

[8] Benvenga S, Toscano A, Rodolico C, Vita G, Trimarchi F (2001). Endocrine evaluation for muscle pain. J R Soc Med.

[9] Katzberg HD, Kassardjian CD (2016). Toxic and endocrine myopathies. Continuum (Minneap Minn).

[10] Ladenson PW, Singer PA, Ain KB, Bagchi N, Bigos ST, Levy EG (2000). American Thyroid Association guidelines for detection of thyroid dysfunction. Arch Intern Med.

[11] Rooyackers OE, Nair KS (1997). Hormonal regulation of human muscle protein metabolism. Annu Rev Nutr.

[12] McKeran RO, Slavin G, Andrews TM, Ward P, Mair WG (1975). Muscle fibre type changes in hypothyroid myopathy. J Clin Pathol.

[13] Norris FH, Panner BJ (1966). Hypothyroid myopathy. Clinical, electromyographical, and ultrastructural observations. Arch Neurol.

[14] Mastaglia FL, Ojeda VJ, Sarnat HB, Kakulas BA (1988). Myopathies associated with hypothyroidism: a review based upon 13 cases. Aust N Z J Med.

[15] Khaleeli AA, Gohil K, McPhail G, Round JM, Edwards RH (1983). Muscle morphology and metabolism in hypothyroid myopathy: effects of treatment. J Clin Pathol.

[16] Caiozzo VJ, Haddad F (1996). Thyroid hormone: modulation of muscle structure, function, and adaptive responses to mechanical loading. Exerc Sport Sci Rev.

[17] Wład H, Fenrych W, Lacka K, Sikorska-Horst W (1988). Urinary glycosaminoglycans in patients with hypothyroidism and in healthy subjects. J Clin Chem Clin Biochem.

[18] Torres CF, Moxley RT (1990). Hypothyroid neuropathy and myopathy: clinical and electrodiagnostic longitudinal findings. J Neurol.

[19] Kahaly GJ, Diana T, Glang J, Kanitz M, Pitz S, König J (2016). Thyroid stimulating antibodies are highly prevalent in Hashimoto’s thyroiditis and associated orbitopathy. J Clin Endocrinol Metab.

[20] Bridwell RE, Willis GC, Gottlieb M, Koyfman A, Long B (2021). Decompensated hypothyroidism: a review for the emergency clinician. Am J Emerg Med.

[21] Chaker L, Bianco AC, Jonklaas J, Peeters RP (2017). Hypothyroidism. Lancet.

[22] Rodolico C, Bonanno C, Pugliese A, Nicocia G, Benvenga S, Toscano A (2020). Endocrine myopathies: clinical and histopathological features of the major forms. Acta Myol.

[23] Aarsæther E, Joakimsen R, Halvorsen H, Sildnes T, Sivertsen O, Due J (2020). Hoffmann’s syndrome necessitating forearm fasciotomy: a case report. J Med Case Rep.

[24] Tahir F, Qadar LT, Khan M, Hussain H, Iqbal SU (2019). Hoffmann’s syndrome secondary to pendred syndrome: a rare case. Cureus.

[25] Saïd F, Tliba A, Khanfir M, Lamloum M, Habib Houman M (2018). Hoffmann syndrome : about two new cases. Rev Med Brux.

[26] Aydın H, Fındıklı HA, Tutak AS, Aydın B, Algın A (2017). Muscular hypertrophy as atypical initial presentation of hypothyroidism. Acta Endocrinol (Buchar).

[27] Achappa B, Madi D (2017). Hoffmann’s syndrome—a rare form of hypothyroid myopathy. J Clin Diagn Res..

[28] Chung J, Ahn K-S, Kang CH, Hong S-J, Kim BH (2015). Hoffmann’s disease: MR imaging of hypothyroid myopathy. Skeletal Radiol.

[29] Nalini A, Govindaraju C, Kalra P, Kadukar P (2014). Hoffmann’s syndrome with unusually long duration: report on clinical, laboratory and muscle imaging findings in two cases. Ann Indian Acad Neurol.

[30] Senanayake HM, Dedigama AD, De Alwis RP, Thirumavalavan K (2014). Hoffmann syndrome: a case report. Int Arch Med.

[31] Cebeci AN, Güven A, Saltik S, Mesci C (2013). Hoffmann’s syndrome and pituitary hyperplasia in an adolescent secondary to Hashimoto thyroiditis. J Pediatr Endocrinol Metab.

[32] Praveen KAS, Aslam S, Dutta TK (2011). HoffMann’s syndrome: a rare neurological presentation of hypothyroidism. Int J Nutr Pharmacol Neurol Dis..

[33] Tuncel D, Cetinkaya A, Kaya B, Gokce M (2008). Hoffmann’s syndrome: a case report. Med Princ Pract.

[34] Kaux J-F, Castermans C, Delmotte P, Bex M (2007). Hoffmann syndrome presenting to the emergency department. Ann Readapt Med Phys.

[35] Ozdag MF, Eroglu E, Ulas UH, Ipekdal I, Odabasi Z, Vural O (2005). Early diagnosis and treatment reverse clinical features in Hoffmann’s syndrome due to hypothyroid myophaty: a case report. Acta Neurol Belg.

[36] Aleem MA, Paramasivam M, Samson TA (2004). Hoffmann syndrome. J Assoc Physicians India.

[37] Qureshi W, Hassan G, Khan GQ, Kadri SM, Kak M, Ahmad M (2005). Hoffmann’s syndrome: a case report. Ger Med Sci.

[38] Vasconcellos LFR, Peixoto MC, de Oliveira TN, Penque G, Leite ACC (2003). Hoffman’s syndrome: pseudohypertrophic myopathy as initial manifestation of hypothyroidism. Case report. Arq Neuropsiquiatr.

[39] Sidibe EH, Diop AN, Thiam A, Diagne PM, Sarr A, Toure M (2001). Hoffmann’s syndrome in hypothyroid myopathy. Report of a case in an African. Jt Bone Spine..

[40] Klein I, Parker M, Shebert R, Ayyar DR, Levey GS (1981). Hypothyroidism presenting as muscle stiffness and pseudohypertrophy: Hoffmann’s syndrome. Am J Med.

[41] Horak HA, Pourmand R (2000). Endocrine myopathies. Neurol Clin.

[42] Barahona MJ, Mauri A, Sucunza N, Paredes R, Wägner AM (2002). Hypothyroidism as a cause of rhabdomyolysis. Endocr J.

[43] Hekimsoy Z, Oktem IK (2005). Serum creatine kinase levels in overt and subclinical hypothyroidism. Endocr Res.

[44] Eslamian F, Bahrami A, Aghamohammadzadeh N, Niafar M, Salekzamani Y, Behkamrad K (2011). Electrophysiologic changes in patients with untreated primary hypothyroidism. J Clin Neurophysiol.

[45] de Krom MC, Kester AD, Knipschild PG, Spaans F (1990). Risk factors for carpal tunnel syndrome. Am J Epidemiol.

[46] Duyff RF, Van den Bosch J, Laman DM, van Loon BJ, Linssen WH (2000). Neuromuscular findings in thyroid dysfunction: a prospective clinical and electrodiagnostic study. J Neurol Neurosurg Psychiatry.

[47] Ono S, Inouye K, Mannen T (1987). Myopathology of hypothyroid myopathy. Some new observations. J Neurol Sci.

[48] Jonklaas J (2022). Role of levothyroxine/liothyronine combinations in treating hypothyroidism. Endocrinol Metab Clin N Am.

