# Supplementary material for: Hoffmann’s syndrome in the differential work-up of myopathic complaints: a case report
Source: J Med Case Rep. 2023 Oct 31;17:473. doi: 10.1186/s13256-023-04184-6 (PMC10617199; doi:10.1186/s13256-023-04184-6)
Supplement: Supplementary file 1 — Additional file 1: Table A1. Case reports of Hoffmann’s syndrome. [file 13256_2023_4184_MOESM1_ESM.pdf]

Additional Table A1. Case reports of Hoffmann's syndrome.

Reference	Patient	Symptoms	Relative elevation of CK and TSH	Clinical neurophysiology	Imaging
Aarsæther et al., 2020 [23]	41 years, male, Hypothyroidism after radiotherapy	Pain in the swollen right forearm, pain of the calves	CK 6fold<br>TSH 7fold		
Tahir et al., 2019 [24]	28 years, male, Pendred syndrome	Generalized upper and lower limb weakness along with progressive facial puffiness, lower limb swelling	CK 6.5 fold<br>TSH 8.8 fold	**EMG** and **NCS** normal	**Muscle biopsy:** changes in fibre size and local fibrosis
Said et al., 2018 [25]	49 years, male	Periorbital edema, muscle cramps, muscular hypertrophy	CK 2fold<br>TSH 16.5fold	**EMG:** myopathic changes	
	57 years, male	Muscle cramps, muscular hypertrophy	CK 22fold<br>TSH 17.5fold		
Aydin et al., 2017 [26]	24 years, male, Hashimoto thyreoiditis	Swelling and pain in both calf muscles, painful muscle cramps, weakness, forgetfulness and weight gain, hypertrophic calf muscles	CK 11fold<br>TSH 5fold		
Achappa & Madi, 2017 [27]	45 years, male	Pedal edema, swelling of the face, macroglossia, hypertrophy of calf muscles, mild proximal lower limb weakness	CK 56fold<br>TSH 69.5fold		
Chung et al., 2015 [28]	34 years, male	Pain, muscle stiffness and tenderness in both lower limbs, orbital swelling and a puffy face	CK 37fold<br>Myoglobin 3fold<br>TSH 24.5fold	**EMG:** widespread abnormal spontaneous activity without neuropathic potentials	**MRI:** hypertrophic lower extremity muscles with gyrus-like configuration. T2 hyperintensity in the bilateral medial head of the gastrocnemius and scattered, patchy T2 hyperintensities in vastus and semimembranosus muscles with slight gadolinium enhancement. **Muscle biopsy:** no endomysial lymphocytic infiltration. Focal some angulated type I fibre atrophy

Lee et al. 2015 [3]	34 years, male	Progressive leg swelling and pain, lethargy, intolerance to cold, preorbital swelling, muscle weakness, muscle hypertrophy	CK 37fold<br>Myoglobin 3.3 fold<br>TSH 24.7 fold	**NCS:** bilateral carpal tunnel syndrome<br>**EMG:** mild spontaneous activity and polyphasic MUAP with low amplitude	**MRI:** symmetric multifocal patchy muscle enhancements with diffuse muscle swelling in thighs and lower legs, patchy T2-hyperintensity in gastrocnemius muscles
Nalini et al., 2014 [29]	35 years, male	Progressive dysarthria, progressive calf hypertrophy and stiffness of lower limbs with proximal muscle weakness	CK 35fold<br>TSH 18fold	**NCS:** mildly prolonged distal latencies and reduced compound muscle action potentials with mildly reduced conduction velocity	**MRI:** T1- hypertrophy of and T2-hyperintensity of the muscles of the legs
	24 years, male	Myalgia and progressive generalized muscle hypertrophy, cramps	CK 12.5fold<br>TSH 18fold	**EMG:** short duration small polyphasic MUAPs. No pseudomyotonic discharges<br>**NCS:** normal	
Senanayake et al., 2014 [30]	39 years, male, primary hypothyroidism	Macroglossia, hypertrophy of calf muscles, proximal upper and lower limb muscle weakness, generalized hyporeflexia	CK 5.5fold<br>TSH 6.3fold	**EMG:** low amplitude and short duration MUAPs with early recruitment	
Cebeci et al., 2013 [31]	12 years, male, Hashimoto thyroiditis	Hypertrophy in the shoulder and calf muscles, muscle pain, headache, fatigue, proximal muscle weakness	CK 2.7fold<br>TSH 24fold	**EMG** normal	
Praveen et al., 2011 [32]	40 years, male	Predominant proximal muscular weakness, mild periorbital puffiness, macroglossia, dry coarse skin, hoarse voice, hypertrophy of calf muscles	CK 6.4fold<br>TSH 20fold	**NCS:** normal.<br>**EMG:** myopathic MUAPs with small amplitude and duration.	
Tuncel et al., 2008 [33]	24 years, male, autoimmune chronic thyroiditis	Fatigue and motor weakness and stiffness in lower extremities, muscle cramps, swelling of face and shoulders, hypertrophy of calves, proximal muscle weakness	CK 35.7fold<br>TSH 23.8fold	**EMG:** reduced amplitude and duration of MUAPs	
Kaux et al., 2007 [34]	31 years, male	Asthenia, muscular pain, cramps, joint pain, muscle hypertrophy	CK 40fold<br>TSH 25fold	**EMG:** fibrillations, small and polyphasic MUAPs	

Ozdag et al., 2005 [35]	22 years, male, primary hypothyroidism	Progressive weakness in arms and legs, muscle cramps and pain, muscular hypertrophy	CK 38fold TSH 30fold	**NCS:** bilateral carpal tunnel syndrome **EMG:** normal	**Muscle biopsy:** normal
Udayakumar et al., 2005 [4]	43 years, male	Lower limb weakness and dyspnea, calf muscle hypertrophy	CK 12.7fold TSH 27fold	**NCS:** normal **EMG:** mild spontaneous activity, polyphasic MUAPs with small amplitude and duration	
Aleem et al., 2004 [36]	27 years, female	Hypertrophy of calf muscles, myxedema, diffuse myalgia	CK 2.3 fold TSH 10fold		
Qureshi et al., 2005 [37]	21 years, male	Generalized weakness, cold intolerance, constipation, hoarse voice, progressive enlargement of muscles of thighs and back with crampy pains	CK 8.5fold TSH 2fold	**NCS:** normal **EMG:** complex repetitive discharges	**Muscle biopsy:** hypertrophy of muscle fibres with increased nuclei, few necrotic fibres and mucoid deposits at places
Vasconcellos et al., 2003 [38]	42 years, male	Progressive muscular weakness, cramps, myalgia, macroglossia, hoarse voice, hypertrophy of the lower limbs, hyporeflexia	CK 18.2 fold TSH 23.9fold	**NCS:** normal **EMG:** mixed pattern myogenic and neurogenic changes	
Sidibe et al., 2001 [39]	31 years, female, primary hypothyroidism	Severe weakness in all four limbs, myalgia, swelling of the face, marked hypertrophy of the limb muscles	CK 23.2 fold TSH 7.3 fold	**NCS:** distal axonal neuropathy **EMG:** myogenic pattern	**Muscle biopsy:** perivascular atrophy without inflammation
Torres & Moxley, 1990 [18]	59 years, male	Progressive proximal muscle weakness, muscle pain, cramps, stiffness, fatigue, enlarged calves, percussion myotonia	CK 48.4fold TSH not mentioned	**NCS:** prolonged distal motor latencies, mildly slow motor conduction velocity, loss of sensory nerve action potentials. **EMG:** complex repetitive discharges, low amplitude, short, polyphasic MUAPs	**Muscle biopsy:** few necrotic muscle fibres, phagocytosis, regenerating fibres, increased internal nuclei and increased variation in fibre size

Klein et al., 1981 [40]	56 years, female	Progressive muscular stiffness, cramps, lethargy, weight gain, periorbital edema, muscular weakness, muscular hypertrophy	CK 10.5fold<br>TSH 10fold	**EMG:** increased insertional activity, fibrillations and PSW, decreased recruitment	
	54 years, male	Muscular weakness, weight gain, muscle stiffness, ataxic gait, increase in muscle size	CK 27.8 fold<br>TSH 6.5fold		

Abbreviations are: creatine kinase, CK; electromyography, EMG; magnetic resonance imaging, MRI; motor unit action potential, MUAP; nerve conduction study, NCS; thyroid stimulating hormone, TSH.
